# Single Sessions of High-Definition Transcranial Direct Current Stimulation Do Not Alter Lower Extremity Biomechanical or Corticomotor Response Variables Post-stroke

**DOI:** 10.3389/fnins.2019.00286

**Published:** 2019-04-11

**Authors:** John Harvey Kindred, Steven A. Kautz, Elizabeth Carr Wonsetler, Mark Goodman Bowden

**Affiliations:** ^1^Ralph H. Johnson, Veteran’s Administration Medical Center, Charleston, SC, United States; ^2^Division of Physical Therapy, College of Health Professions, Medical University of South Carolina, Charleston, SC, United States; ^3^Department of Health Sciences and Research, College of Health Professions, Medical University of South Carolina, Charleston, SC, United States; ^4^Department of Physical Therapy, School of Health Sciences, High Point University, High Point, NC, United States

**Keywords:** HD-tDCS, anodal, cathodal, gait, mobility, tDCS, brain stimulation, TMS

## Abstract

Transcranial direct current stimulation (tDCS) is a non-invasive brain stimulation technique used to modulate cortical activity. However, measured effects on clinically relevant assessments have been inconsistent, possibly due to the non-focal dispersion of current from traditional two electrode configurations. High-definition (HD)-tDCS uses a small array of electrodes (*N* = 5) to improve targeted current delivery. The purpose of this study was to determine the effects of a single session of anodal and cathodal HD-tDCS on gait kinematics and kinetics and the corticomotor response to transcranial magnetic stimulation (TMS) in individuals post-stroke. We hypothesized that ipsilesional anodal stimulation would increase the corticomotor response to TMS leading to beneficial changes in gait. Eighteen participants post-stroke (average age: 64.8 years, *SD*: 12.5; average months post-stroke: 54, *SD*: 42; average lower extremity Fugl-Meyer score: 26, *SD*: 6) underwent biomechanical and corticomotor response testing on three separate occasions prior to and after HD-tDCS stimulation. In a randomized order, anodal, cathodal, and sham HD-tDCS were applied to the ipsilesional motor cortex for 20 min while participants pedaled on a recumbent cycle ergometer. Gait kinetic and kinematic data were collected while walking on an instrumented split-belt treadmill with motion capture. The corticomotor response of the paretic and non-paretic tibialis anterior (TA) muscles were measured using neuronavigated TMS. Repeated measures ANOVAs using within-subject factors of time point (pre, post) and stimulation type (sham, anodal, cathodal) were used to compare effects of HD-tDCS stimulation on measured variables. HD-tDCS had no effect on over ground walking speed (*P* > 0.41), or kinematic variables (*P* > 0.54). The corticomotor responses of the TA muscles were also unaffected by HD-tDCS (resting motor threshold, *P* = 0.15; motor evoked potential (MEP) amplitude, *P* = 0.25; MEP normalized latency, *P* = 0.66). A single session of anodal or cathodal HD-tDCS delivered to a standardized ipsilesional area of the motor cortex does not appear to alter gait kinematics or corticomotor response post-stroke. Repeated sessions and individualized delivery of HD-tDCS may be required to induce beneficial plastic effects. Contralesional stimulation should also be investigated due to the altered interactions between the cerebral hemispheres post-stroke.

## Introduction

Less than 50% of individuals post-stroke return to independent community ambulation (Jorgensen et al., 1995) and 73% have some type of long term disability (Gresham et al., 1995). Brain plasticity, i.e., structural and functional circuit reorganization, occurs after stroke and during motor rehabilitation (Sampaio-Baptista et al., 2018; Zhao and Willing, 2018). In some cases, plasticity can be beneficial to recovery. However, maladaptive processes can also occur leading to prolonged recovery or disability. Therefore, enhancing the positive effects of adaptive neuroplasticity and minimizing maladaptive plasticity are integral in providing adequate post-stroke rehabilitation.

Non-invasive brain stimulation techniques have been proposed to enhance the beneficial plastic effects of post-stroke rehabilitation (Fregni and Pascual-Leone, 2007). One such technique is transcranial direct current stimulation (tDCS) which involves the application of direct current, usually 1–2 mA, to the scalp over a targeted cortical area (Woods et al., 2016). Combining tDCS and traditional therapies may potentially improve stroke recovery due to Hebbian principals of motor learning (Schlaug and Renga, 2008). A recent review/meta-analysis reported beneficial increases in lower extremity muscular strength and mobility, although no effects on walking speed, walking endurance, or balance function could be detected (Li et al., 2018). While some beneficial clinical effects have been seen with traditional tDCS, imaging and finite element modeling studies demonstrate that current flow may be concentrated in areas away from targeted neuronal populations when using traditional two electrode montages (Datta et al., 2010; Antal et al., 2011). To overcome this lack of specificity, modeling studies predicted that replacing the traditional two electrode setup, typically rectangular electrodes approximately 35 cm^2^, with an array of smaller circular electrodes, <1.2 cm in diameter, can more precisely deliver current (Minhas et al., 2010). Delivering current with a 4 × 1 ring montage, termed High-definition (HD)-tDCS, increases the current density (Caparelli-Daquer et al., 2012). In this montage the central electrode has opposite polarity of the four reference electrodes, i.e., one central anode and four surrounding cathodes or a central cathode with four surrounding anodes. Due to the location of the sensorimotor cortical representation of the lower extremities deep within the cortex along the longitudinal fissure utilizing more targeted current delivery with HD-tDCS may enhance outcomes related to motor rehabilitation and nervous system plasticity in chronic stroke, especially when compared with traditional tDCS.

Studies using HD-tDCS in healthy individuals has shown increases in the corticomotor response and enhanced cross-facilitation in the upper extremities of healthy individuals (Caparelli-Daquer et al., 2012; Cabibel et al., 2018). However, there is currently a lack of information about the effects of HD-tDCS in the lower extremities of people post-stroke, specifically, regarding gait and the corticomotor response to transcranial magnetic stimulation (TMS). Therefore, the purpose of this study was to determine the effects of a single session of HD-tDCS on gait kinetics, kinematics, and lower extremity corticomotor response in chronic stroke. Our *a priori* hypothesis was that anodal HD-tDCS applied to the ipsilesional motor cortex would increase motor evoked potential (MEP) amplitude and decrease resting motor threshold (rMT) and MEP latency of the paretic TA. Furthermore, we expected that with an altered corticomotor response we would see beneficial changes to gait characteristics.

## Materials and Methods

### Ethics Statement

All methods and procedures were approved by the local Institutional Review Board and conformed to the Declaration of Helsinki. All participants signed informed consent prior to their participation in any aspect of the study.

### Participants and Study Procedures

Twenty-one individuals post-stroke were recruited from the Ralph H. Johnson VA Medical Center (VAMC)/Medical University of South Carolina (MUSC) Stroke Recovery Research Center’s (SRRC) participant database. Inclusion criteria for the study were: >6 months post-stroke, able to ambulate at least 10 m with minimal use of assistive devices, have a locomotor control impairment as indicated by scoring less than the maximum, 34, on the lower extremity Fugl-Meyer assessment (Fugl-Meyer et al., 1975), and have a measurable corticomotor response. Prospective participants were invited to the VAMC/MUSC SRRC for a total of four visits: one initial visit and three experimental visits.

### Initial Visit

During the first visit, participants signed informed consent and underwent clinical testing. The clinical tests included: Berg Balance Scale (Berg et al., 1995), Functional Gait Assessment (Wrisley et al., 2004), 6-min walk test, and the lower extremity Fugl-Meyer assessment. Clinical testing was performed by a licensed physical therapist. After clinical testing, participants underwent TMS examination to identify the cortical representation, i.e., hotspot, of the tibialis anterior (TA) on the paretic and non-paretic legs. The TMS assessment was performed using a double-cone coil and a Magstim BiStim2 stimulator (Magstim, Inc., Whitland, United Kingdom). Hotspot identification was guided by neuronavigation (Brainsight, Rogue Research, Montreal, QC, Canada) (Charalambous et al., 2018). Briefly, hotspots were identified using a single suprathreshold single TMS pulse applied over a 3x5 grid transposed over an average Montreal Neurological Institute MRI brain image. Two trials were performed and the spot with the greatest average response, i.e., largest MEP amplitude, was used during the subsequent experimental visits. All MEPs were evoked while the muscle was at rest. If muscle activity was noted prior to the application of the TMS pulse the trial was discarded, and another pulse was applied.

### Experimental Visits

During the experimental visits, visits 2–4, participants underwent biomechanical and neurophysiological testing prior to and post-receiving HD-tDCS. Participants received sham, anodal, or cathodal HD-tDCS stimulation in a randomized order. During HD-tDCS stimulation participants also pedaled on a recumbent cycle ergometer at a self-selected cadence with minimal resistance to activate the cortical tissue receiving the HD-tDCS stimulation.

#### Biomechanical Assessment

Gait kinetics and kinematics were measured using an active LED marker set applied in a modified Helen Hayes setup while participants walked on an instrumented split-belt treadmill with bilateral force plates (Bertec, Corp., Worthington, OH, United States) (Dean et al., 2017). Participants performed two 30 s trials at their preferred self-selected comfortable speed with motion capture. Motion capture data was sampled at 100 Hz using a 12-camera system (PhaseSpace, San Leandro, CA, United States). Ground reaction forces (GRFs) were sampled at 1000 Hz. After treadmill walking participants performed two trials of over ground walking on a GAITRite electronic walkway (CIR Systems, Inc., Franklin, NJ, United States) at their preferred comfortable walking speed. Participants wore a harness attached to the ceiling to mitigate their chance of falling during all walking tests.

#### Corticomotor Response Measurement

The assessment of the corticomotor response to TMS was initiated within 10 min of completion of the walking trials. The corticomotor response, measured as a MEP, was recorded using surface EMG (sEMG). Electrodes were placed over the TA muscle bellies and signals were amplified 1000x and recorded at 5000 Hz using Signal 6.03 (Cambridge Electronic Design, Cambridge, United Kingdom) for offline analysis. After the participant was seated comfortably in an adjustable chair with sEMG electrodes over the paretic and non-paretic TA muscles, measurement of the rMT commenced using parameter estimation by sequential testing (PEST) (Mishory et al., 2004). To determine the rMT two PEST procedures were performed using the hotspot identified during the initial visit. The anatomical location of the hotspot was maintained between visits by utilizing neuronavigation. The PEST program directed the intensity of the TMS stimuli applied to the muscle’s hotspot. The measurement of the rMT was performed twice, and the average of the two trials was used as the participant’s rMT for that experimental session. Once the rMT was established for each muscle, 10 single TMS pulses were applied at 120% of the rMT to the paretic and non-paretic TA hotspots using a double cone coil while the muscles were at rest.

#### HD-tDCS Stimulation

**FIGURE 1:**
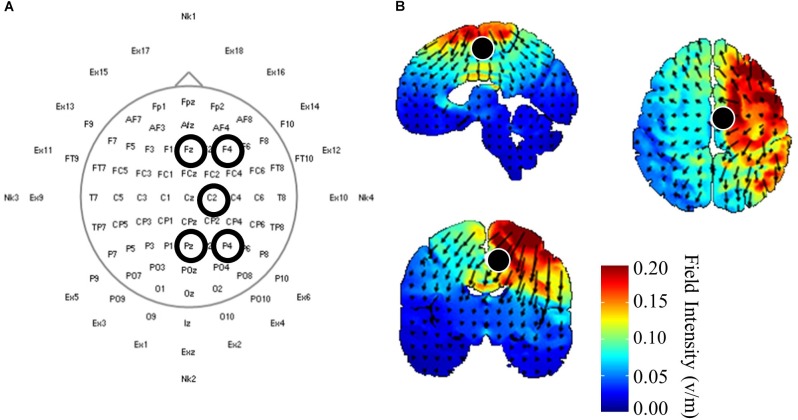
High-definition (HD) transcranial direct current application and modeling. **(A)** HD-tDCS electrode placements according to standard 10–10 nomenclature. The central electrode was placed at C_2_ with surrounding reference electrodes located at F_z_, F4, P_z_, P4 for participants with strokes in the right hemisphere. Left hemisphere stroke locations were C1, F_z_, F3, P_z_ P3. **(B)** HD-tDCS current flow modeled using HDExplore (Soterix Medical, Inc.). Modeling estimated a field intensity of approximately 1 v/m at the right paracentral lobule, which is the location of the lower extremity sensorimotor representation within the cortex. Cathodal modeling showed a similar field intensity but with current moving in opposite directions.

Once initial gait and corticomotor response measurements were completed, participants received 20 min of one of three HD-tDCS (Soterix Medical, Inc., New York, NY, United States) stimulations: central anodal, central cathodal, or sham. Participants received the stimulations in a randomized order over the three experimental visits, and both participants and investigators were blinded the stimulation protocol used during that session. Electrodes were held in place using a cap with a pre-defined grid utilizing the standard 10–10 system (Chatrian et al., 1985). The central HD-tDCS electrode was positioned on either C1 or C2, whichever corresponded with the ipsilesional motor cortex (Figure 1A). This location was chosen from modeling performed using HDExplore (Soterix Medical, Inc.) to deliver current to the ipsilesional M1 with minimal current delivered to the contralesional cortex (Figure 1B). The HDExplore program used a standard MRI image to model current flow, stroke specific modeling was not available. A current of 2 mA was applied through the central electrode during anodal conditions that was returned through the four reference electrodes. During cathodal stimulation 0.5 mA was delivered through each of the four reference electrodes and returned through the central electrode. The sham condition was similar to anodal stimulation, however the HD-tDCS stimulator automatically reduced the current to zero after 30 s. To activate the motor cortex while receiving HD-tDCS participants pedaled on a recumbent cycle ergometer (Monark Exercise AB, Vansbro, Sweden) at a self-selected pace with minimal resistance. After stimulation was concluded participants immediately underwent reassessment of the corticomotor response followed by biomechanical assessment. Post-biomechanical and corticomotor response testing was conducted using the same parameters as pre-testing, e.g., equal treadmill speeds and TMS stimulator output intensity.

### Data Analyses

#### Gait Kinetics and Kinematics Analyses

Motion capture data were low-pass filtered at 10 Hz using a 4^th^ order, zero-lag Butterworth filter. Body segment kinematics were determined using a custom model (Visual3D, Germantown, MD, United States). Segment COM locations were calculated using anthropometric and inertial properties (de Leva, 1996). Kinetic and kinematic variables included: anterior (propulsive) GRFs, posterior (braking) GRFs, ankle power, ankle work, cadence, paretic and non-paretic step length. All post-stimulation kinematic and kinetic variables were collected at speeds matched to pre-testing speeds on the instrumented treadmill.

#### MEP Analysis

**FIGURE 2:**
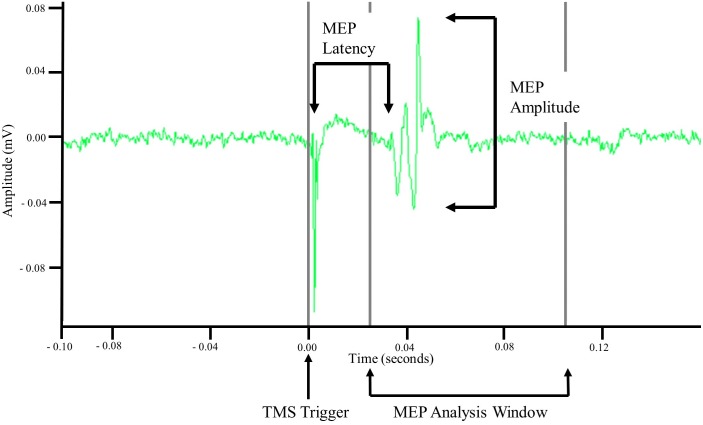
Example motor evoked potential (MEP) recorded via surface electromyography. Unprocessed surface electromyographic signal recorded after a single TMS pulse was applied to cortical representation of the tibialis anterior (TA) muscle. MEP amplitude was measured as the difference in minimal and maximal amplitude of the signal within an 0.08 s analysis window that began 0.025 s after application of the TMS pulse. MEP latency was measured as the time between the TMS pulse and the initiation of the MEP.

Motor evoked potentials were recorded via sEMG. Using the TMS pulse trigger, a 0.5 s data window was recorded starting 0.1 s before the TMS trigger. Offline analyses of recorded EMG data were performed in MATLAB R2017b (MathWorks, Natick, MA, United States). Data were imported into MATLAB and demeaned using the average signal of the first and last 0.05 s. MEP amplitude was then calculated as the difference between the maximum and minimum values in a 0.08 s window starting at 0.025 s. Once amplitude was calculated the signal was rectified and the time from the TMS trigger pulse to the start of the MEP, i.e., latency, was measured. MEP latency was defined as the point when the rectified MEP signal amplitude was greater than the mean plus three standard deviations of the signal amplitude occurring 0.08 s before the TMS trigger pulse (Cacchio et al., 2011; Charalambous et al., 2018) for at least 0.001 s. Latency was then normalized to participant height and is reported as ms/m. Once all MEP metrics were calculated the data were exported and visually inspected to ensure the accuracy and validity of the values. For MEPs to be considered valid the following criteria had to be met: an amplitude greater than 50 μV and a non-normalized latency between 0.025 and 0.105 s. Stimulation trials that did not evoke at least 4/10 MEPs were not used for analysis. An example MEP is displayed in Figure 2.

### Statistical Analysis

All data are reported as Mean and Standard Deviation (SD) unless otherwise noted. Variables that were comprised of values from both legs (walking speed, cadence) were analyzed using a two-factor Repeated Measures ANOVA using within-subject factors of stimulation type (sham, anodal, cathodal) and time point (pre-stimulation, post-stimulation). Additional variables that that were recorded for each leg/side (step length, GRFs, rMT, MEP amplitude, MEP normalized latency), were analyzed using a three-factor Repeated Measures ANOVA with within-subject factors of: stimulation type (sham, anodal, cathodal), leg (paretic, non-paretic), and time point (pre-stimulation, post-stimulation). Sphericity of the models was tested using Mauchly’s Test of Sphericity, and in the case of significant findings Greenhouse–Geiser corrected *P-values* are reported. In all others the sphericity was assumed. When a significant *F*-test was present *post hoc* comparisons were made using Bonferroni corrections. Alpha was set to 0.05. All statistical analysis was performed using IBM SPSS Statistics for Windows, Version 24 (IBM, Corp., Somers, NY, United States).

## Results

### Sample Demographics

Two individuals failed to screen into the study (no locomotor control impairment as assessed by achieving the maximum Fugl-Meyer score) and one participant was removed because they had no discernable corticomotor response (no MEPs elicited in the paretic or non-paretic TA) leaving a final sample of 18. The average age was 64.8 (*SD:* 12.5) years with time post-stroke of 53.7 (*SD:* 42.1) months. The average lower extremity Fugl-Meyer score was 26 (*SD*: 6) and 10 (*SD*: 3) for motor and sensory evaluations respectively. Other demographic variables are listed in Table 1.

**Table 1:** Demographic variables.

Ht. (m)	Wt. (kg)	BMI	BBS	FGA	6MWT (m)
1.7	185.9	29.5	Median 50	Median 19	347.5
*SD*: 0.1	*SD*: 37.8	*SD*: 7.9	Range: 25–56	Range: 9–30	*SD*: 97.6

*Demographic variables of the sample. Data are reported as Mean and Standard Deviation (SD) unless otherwise noted. BMI, Body Mass Index; BBS, Berg Balance Scale; FGA, Functional Gait Assessment; 6MWT, Six Minute Walk Test.*

### Gait Kinematic Data

**FIGURE 3:**
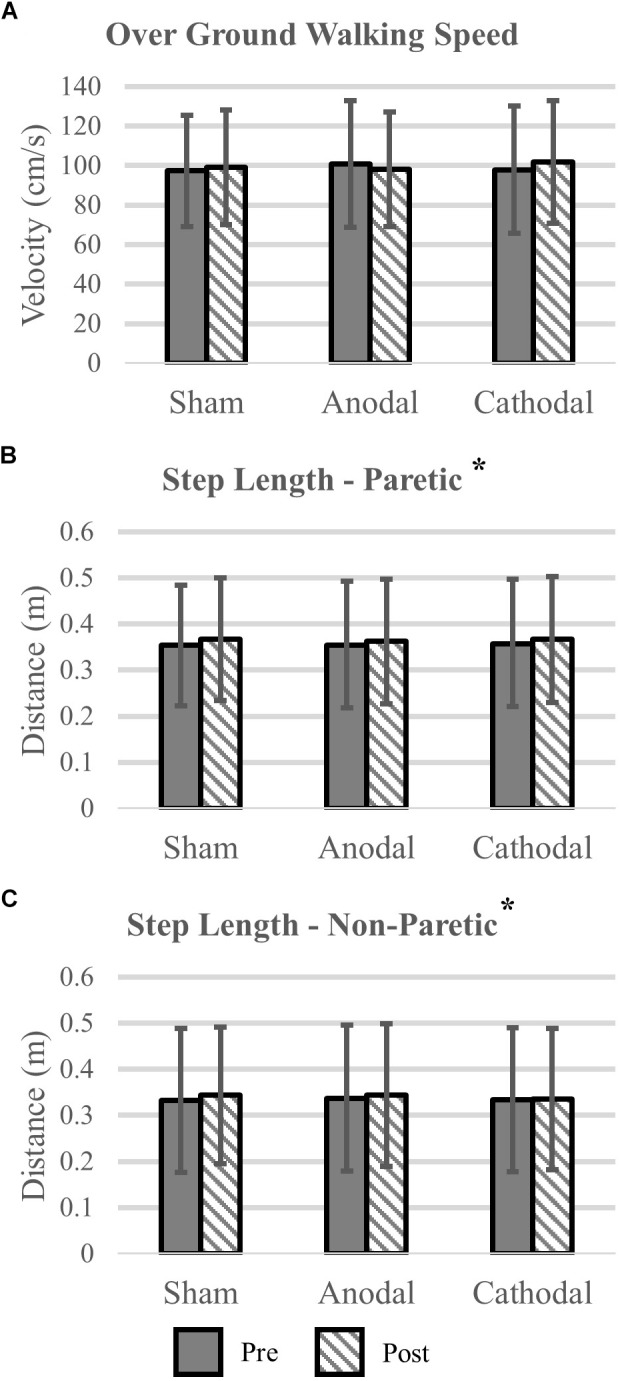
Kinematic variables. **(A)** Over ground walking speeds were not affected by the application of HD-tDCS. **(B,C)** The step length of the paretic and non-paretic legs was increased after HD-tDCS stimulation. However, this increase was not different between the three stimulation types (Sham, Anodal, Cathodal). ^∗^*P* < 0.05.

Gait variables were analyzed for all participants (*N* = 18). Self-selected over ground walking speed, as measured via the GAITRite, did not change after HD-tDCS stimulation (*P* > 0.19). Motion capture revealed that paretic and non-paretic step length was increased after HD-tDCS stimulation (*P* = 0.05); however, the increase was not different between the different stimulation types (*P* = 0.99). Concomitant with the increase in step length, cadence was reduced after HD-tDCS stimulation (*P* = 0.02) and independent of stimulation type (*P* = 0.91). Means and SDs for all variables are displayed in Figure 3 and all *F-*statistics and *P*-values are listed in Table 2.

**Table 2:** Spatial-temporal gait variables statistics.

	Main effect of stimulation type	Main effect time point	Main effect of leg	Interaction(s)
Over ground walking speed	*F* = 0.31 *P* = 0.74	*F* = 0.71 *P* = 0.41	N/A	*F* = 1.73 *P* = 0.19
Cadence	*F* = 0.09 *P* = 0.91	***F* = 6.32** ***P* = 0.02**	N/A	*F* = 0.55 *P* = 0.58
Step length	*F* = 0.01 *P* = 0.99	***F* = 4.58** ***P* = 0.05**	*F* = 1.55 *P* = 0.23	*F* < 0.63 *P* > 0.54

*Bold denotes P < 0.05.*

### Gait Kinetic Data

#### Ground Reaction Forces

**FIGURE 4:**
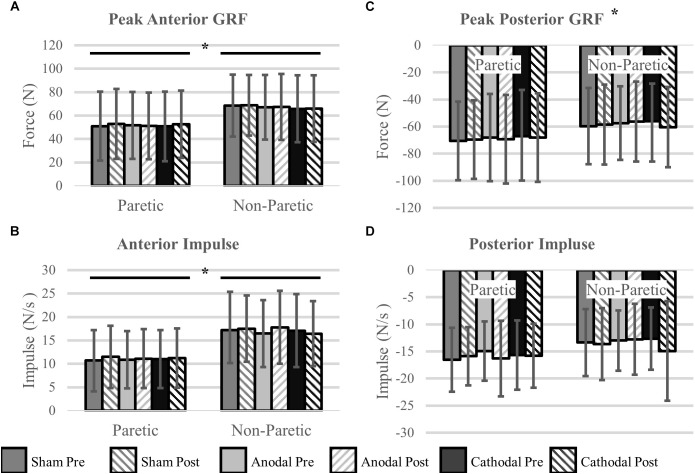
Ground reaction forces. **(A,B)** Maximal propulsive (anterior) GRFs and impulses were not affected by HD-tDCS. The propulsive GRFs and impulses were greater in the non-paretic side compared to the paretic side, which is commonly seen in this population. **(C,D)** Maximal braking (posterior) GRFs and the posterior impulses were not different between the legs. However, there was a significant stimulation type × time point interaction revealing an increase (more negative) in peak braking forces after cathodal stimulation **(C)**. ^∗^*P* < 0.05.

Peak Propulsive (anterior direction) forces and impulse were greater in the non-paretic limb (*P* < 0.02). Peak braking (posterior direction) forces and impulses were not different between the legs (*P* > 0.14). Propulsive GRFs were not changed with the intervention (*P* > 0.32). However, there was a stimulation type × time point interaction with the peak braking GRF (*P* < 0.01). The interaction revealed an increase, more negative, in peak braking forces after cathodal stimulation. No other effects of HD-tDCS were detected for any other kinetic variables (*P* > 0.07). Means and SDs for all variables are displayed in Figure 4 and Table 3 contains all *F*-statistics and *P*-values.

**Table 3:** Kinetic gait variables statistics.

	Main effect of stimulation type	Main effect time point	Main effect of leg	Interaction(s)
Peak anterior GRF	*F* = 0.15 *P* = 0.87	*F* = 1.07 *P* = 0.32	***F* = 9.98** ***P* < 0.01**	*F* < 0.73 *P* > 0.49
Anterior GRF impulse	*F* = 0.20 *P* = 0.82	*F* = 1.06 *P* = 0.32	***F* = 7.06** ***P* = 0.02**	*F* < 1.54 *P* > 0.23
Peak posterior GRF	*F* = 0.31 *P* = 0.74	*F* = 0.62 *P* = 0.44	*F* = 2.46 *P* = 0.14	***F* = 8.11** ***P* < 0.01**
Posterior GRF impulse	*F* = 0.72 *P* = 0.50	*F* = 2.11 *P* = 0.17	*F* = 2.08 *P* = 0.17	*F* < 3.29 *P* > 0.07

*GRF, ground reaction force: a significant stimulation type × time point interaction was detected for cathodal stimulation only. No other interactions were significant (F < 1.65, P > 0.21). Bold denotes P < 0.05.*

#### Ankle Power and Work

**FIGURE 5:**
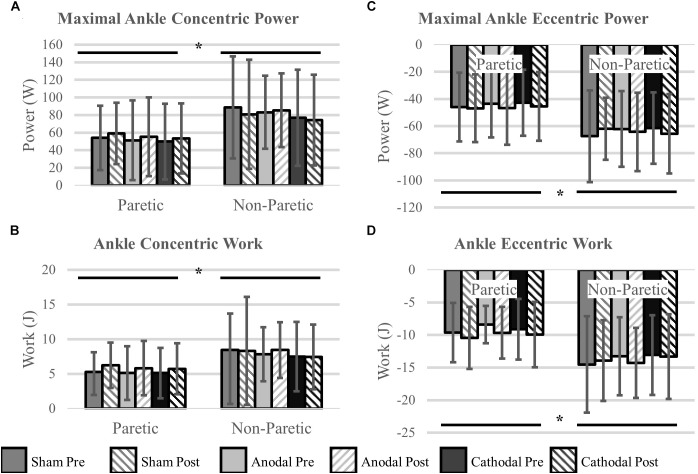
Ankle power and work. **(A,B)** Concentric power and work were unaffected by HD-tDCS but were greater in the non-paretic ankle. **(C,D)** Eccentric power and work were unaffected by HD-tDCS but were greater in the non-paretic ankle. ^∗^Main effect of leg (paretic versus non-paretic) *P* < 0.05.

Ankle power and work were greater on the non-paretic side compared to the paretic side (*P* < 0.04). However, the intervention had no effect ankle power or work (*P* > 0.06). There were no interactions for any variables (*P* > 0.07). Means and SDs for each variable are displayed in Figure 5 and all *F-*statistics and *P*-values are listed in Table 4.

**Table 4:** Ankle power and work statistics.

	Main effect of stimulation type	Main effect time point	Main effect of leg	Interaction(s)
Concentric ankle power	*F* = 0.52 *P* = 0.60	*F* = 0.05 *P* = 0.83	***F* = 7.93** ***P* = 0.01**	*F* < 1.80 *P* > 0.20
Eccentric ankle power	*F* = 0.13 *P* = 0.88	*F* = 1.02 *P* = 0.33	***F* = 11.88** ***P* < 0.01**	*F* < 2.38 *P* > 0.11
Concentric ankle work	*F* = 0.51 *P* = 0.61	*F* = 1.90 *P* = 0.19	***F* = 5.01** ***P* = 0.04**	*F* < 0.61 *P* > 0.45
Eccentric ankle work	*F* = 0.83 *P* = 0.45	*F* = 4.01 *P* = 0.06	***F* = 14.17** ***P* < 0.01**	*F* < 1.08 *P* > 0.33

*Bold denotes P < 0.05.*

### Corticomotor Response

We were unable to elicit a response in the paretic TA of two participants, and subsequently they were removed from the analysis (*N* = 16). There were no differences in rMT prior to initiation of the HD-tDCS stimulations (*P* = 0.15). However, the rMT in the paretic leg was greater compared to the non-paretic leg (*P* < 0.01). Twenty minutes of HD-tDCS and cycling did not change rMT (*P* > 0.15).

**FIGURE 6:**
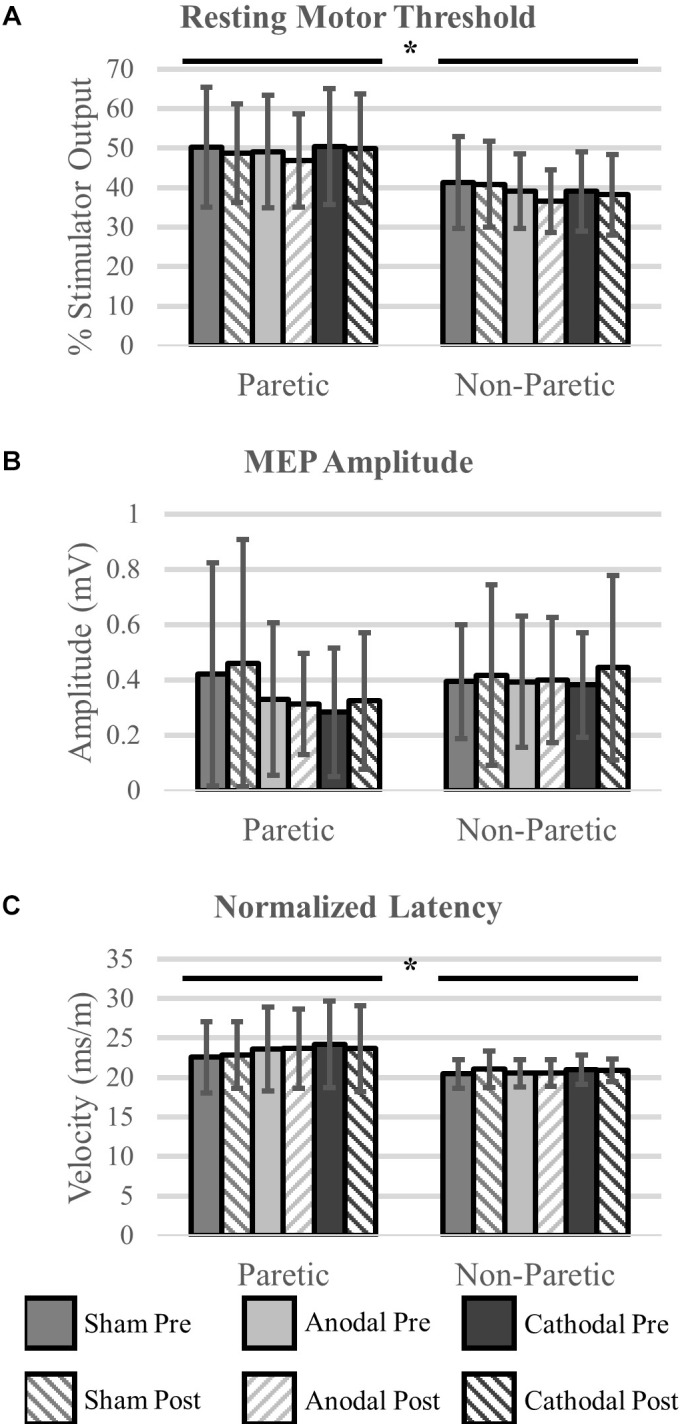
Corticomotor response to single pulse transcranial magnetic stimulation (TMS). A single session of HD-tDCS had no effect on resting motor threshold **(A)**, MEP amplitude **(B)** or latency **(C)**. Similar to previous studies resting motor threshold and latency were greater on the paretic side compared to the non-paretic side. ^∗^Main effect of leg (paretic versus non-paretic) *P* < 0.05.

Out of 2160 single TMS pulses, 1888 (87%) valid MEPs were evoked. Due to some trials not having the required number of valid MEPs (>4) the sample size for MEP amplitude and normalized latency comparisons were reduced to *N* = 12. MEP amplitude was not affected by HD-tDCS (*P* > 0.25) and amplitude was similar between the legs (*P* = 0.60). Normalized MEP latency of the non-paretic side was shorter compared to the paretic side (*P* = 0.05), and HD-tDCS did not affect latency values (*P* > 0.18). No interactions between the variables were identified (*P* > 0.18). Corticomotor response data are displayed in Figure 6 and *F-*statistics and *P*-values are listed in Table 5.

## Discussion

The aim of this study was to identify the effects of HD-tDCS applied over ipsilesional M1 in chronic stroke. We applied three stimulation types (anodal, cathodal, and sham) to 18 participants and compared gait kinetics/kinematics and corticomotor responses pre-/post-stimulation. Contrary to our hypothesis, we were unable to detect any changes in lower extremity kinetic, kinematic, or corticomotor response variables after anodal HD-tDCS. Although, we did detect an increase in peak braking (posterior) GRFs after cathodal stimulation compared to anodal and sham. During our experiments, we did confirm previous reports of differences in kinetic, kinematic, and corticomotor response variables between the paretic and non-paretic lower extremities (Bowden et al., 2006; Allen et al., 2011; Cacchio et al., 2011). Our results seem to suggest that a single session of anodal HD-tDCS using a 4 × 1 montage centered on ipsilesional motor cortex (central electrode = C_2_, reference electrodes = F_z_, F_4_, P_z_, P_4_) does not modulate M1 cortical activity of the lower extremities in chronic stroke.

During gait, when the heel strikes the ground a posterior GRF is applied to slow the forward progress of the body’s center of mass. In this experiment we observed that the peak braking forces were increased after cathodal stimulation. However, total braking force as indicated by the posterior impulses were not different. We were also unable to detect any clinical relevant changes, in terms of gait kinematics, to accompany the increase in peak braking force. This finding leads us to believe that HD-tDCS may have effects on post-stroke gait but the clinical significance and the ability to leverage this change in a meaningful way are still unknown.

Tahtis et al. (2014) and Montenegro et al. (2016) examined the effects of a single session of bihemispheric traditional tDCS on the lower extremities in chronic stroke participants. Both reported some beneficial effects, improved timed up-and-go and force steadiness. However, they had relatively small sample sizes (N ≤ 10) and neither identified a possible mechanism for improvement. In our study we were unable to identify any differences between the stimulation types using a within-subjects method for biomechanical and corticomotor response variables using a slightly larger sample size. Another key difference, besides traditional two-electrode versus HD-tDCS, is that in the previous studies a cathodal stimulation was applied to the contralesional side, due to the placement of the anode over ipsilesional M1 and cathode placed over contralesional M1. It is possible that the effects seen in these earlier studies were due to a reduction in intercortical inhibition originating from the contralesional side by the cathodal stimulation. It is often seen that intercortical inhibition originating from the contralesional hemisphere is often increased post-stroke (Murase et al., 2004; Feng et al., 2013). To further demonstrate the effects of HD-tDCS on intercortical communication, Cabibel et al. (2018) showed that application of HD-tDCS to upper extremity cortical hotspots can enhance cross-facilitation, increasing excitability of unstimulated areas. Our modeling of electrode placement minimized contralesional current flow but may not have been strong enough to modulate the lesioned M1. It may be that in chronic stroke, maladaptive effects of increased intercortical inhibition originating from the contralesional hemisphere may override HD-tDCS to the ipsilesional hemisphere. Applying HD-tDCS to the contralesional M1 may provide a greater effect and requires future study. Measuring lower extremity intercortical communication in chronic stroke may allow for better targeting of current in future HD-tDCS applications.

The brain is highly connected through a network of distinct and identifiable circuits. To compensate for damaged circuits, functional and structural remodeling occurs. The extent of damage and how circuits have reorganized can have an impact in the responsiveness to brain stimulation techniques. Diekhoff-Krebs et al. (2017) reports that individual differences in network connectivity affect the response to repetitive TMS in stroke patients. They showed that participants with greater connectivity between M1 and supplemental motor areas responded to a higher degree to intermittent theta-burst stimulation. Using tDCS in healthy individuals, Rosso et al. (2014b) showed that quicker picture naming was associated with increased volume of the fiber tracts connecting the Broca’s area to the supplemental motor area, as well as an increased functional connectivity between the two regions. One study of tDCS in post-stroke aphasia also indicated the anatomical location of the lesions modified the effectiveness of the stimulation (Rosso et al., 2014a). In that study, cathodal tDCS was applied to the right Broca’s area in participants with lesions located within the left Broca’s area, or another location within the left hemisphere. Participants with lesions located within the left Broca’s area improved more than the group with locations outside that area. Depending on the innate connectivity of an individual, applying HD-tDCS to other nodes within the motor network may prove more effective. As previously mentioned, standard two-electrode tDCS montages delivers current between the anode and cathode electrodes. This may result in greater network modulation compared to HD-tDCS which likely delivers current specifically to one node. Directing HD-tDCS stimulation to different network nodes or altering the montage may lead to greater effects than those seen in this investigation. These nodes may include pre-motor areas or the cerebellum which have strong connections within the motor circuit. Modulating these areas may prove to be useful when trying to improve motor performance in chronic stroke compared to targeted M1 stimulation.

### Limitations

One of the possible reasons for the ineffectiveness of HD-tDCS to modulate the corticomotor response or biomechanical variables in this study may have been due to our stimulation location. Prior to our study, we modeled the delivery of HD-tDCS current to the TA representation of ipsilesional M1. However, when neuronavigationally determining a participant’s TA hotspots, rarely was the greatest response recorded at our stimulated location (C1 or C2). It is possible that current was delivered to a less optimal location to detect changes in lower body kinetics, kinematics, and corticomotor response measures. Placing the central electrode directly over the identified hotspot, as has been done previously in the upper extremities (Cabibel et al., 2018), may direct current to a more ideal location. Another important factor to consider is that current flow was modeled using a non-neurologically impaired brain. Due to technical limitations it was not possible to predict individualized current flow for each participant. It is possible that due to remodeling of the underlying neural tissue current was not delivered to an optimal location to facilitate neuromodulatory effects. Future work is certainly justified to determine the best location to deliver current in brains that have undergone motor circuit remodeling. Integrating fMRI to identify the remodeled motor networks may help investigators direct HD-tDCS stimulation to the most appropriate motor network node.

**Table 5:** Corticomotor response variables statistics.

	Main effect of HD-tDCS type	Main effect of time point	Main effect of leg	Interaction(s)
Resting motor threshold	*F* = 2.03 *P* = 0.15	*F* = 2.34 *P* = 0.15	***F* = 11.90** ***P* < 0.01**	*F* < 1.84 *P* > 0.18
MEP amplitude	*F* = 0.72 *P* = 0.50	*F* = 1.46 *P* = 0.25	*F* = 0.30 *P* = 0.60	*F* < 1.16 *P* > 0.33
Normalized latency	*F* = 1.87 *P* = 0.18	*F* = 0.20 *P* = 0.66	***F* = 4.65** ***P* = 0.05**	*F* < 1.20 *P* > 0.32

*MEP, motor evoked potential. Bold denotes P < 0.05.*

During the application of tDCS it is suggested that for effects to become evident the target area needs to be activated (Schlaug and Renga, 2008). To do this we had our participants perform recumbent cycling. However, we did not standardize or measure the forces/activity of the limbs during this exercise. Participants were instructed to cycle at a comfortable pace, but the cortically derived motor output to the paretic limb likely varied participant to participant. In previous experiments performed by members or our research group, it has been shown that activity of the non-paretic limb during cycling can induce rhythmic activity in the paretic limb (Kautz et al., 2006). It is possible that ipsilesional M1 descending drive was insufficient during rhythmic steady-state pedaling for effects of HD-tDCS to be identified. Another limitation of this modality is that there was likely a greater contribution of the quadriceps and hamstring muscles to perform the task compared to the TA. Using task that was more specific to the TA may have engaged the stimulated M1 to a greater degree and allowed for post-stimulation effects to be seen.

## Conclusion

Twenty minutes of HD-tDCS did not alter lower extremity kinematic, kinetic, or corticomotor response variables post-stroke. Several factors likely contributed to this, and include: the number of session performed, stimulation location, and lower limb activity during stimulation. While single session experiments are often used to identify changes in physiological parameters, most researchers agree that multiple session of brain stimulation are usually required for beneficial plasticity to be identified. In the future we plan on performing similar experimental protocols over several sessions to determine if HD-tDCS can have a beneficial impact in post-stroke motor recovery.

Another area that needs further investigation is the relevance of studying MEP amplitude versus latency. MEP latency seems to be a much more stable parameter in healthy and post-stroke individuals (Charalambous et al., 2018). This was also seen in our sample, as there was less variability in latency in the paretic and non-paretic lower extremities compared to amplitude. MEP amplitude can be affected by many different physiological variables that alter electromyographic response such as hydration status, previous muscle activity, and muscle cross talk (Farina et al., 2004). These factors can change with different amounts of activity and comparing MEP amplitudes across days without some type of correction factor, e.g., reporting as a percent of maximal voluntary contraction, is likely inappropriate. Monitoring changes in latency values may prove to be a more valuable outcome measure in future investigations centered on brain stimulation in chronic stroke.

Due to differences in published protocols with tDCS, direct comparisons between traditional two electrode and HD-tDCS are difficult to make. Future studies are needed to determine which modality would most enhance current rehabilitative practices. As stated previously, during bihemispheric stimulation with traditional tDCS current passes through both the ipsilesional and contralesional sides. Alternate HD-tDCS montages may provide bihemispheric stimulation in a more targeted manner and may lead to enhanced motor recovery post-stroke compared to current clinical practices and traditional tDCS. Additionally, greater research into the standardization of HD-tDCS is needed to ensure future research can be performed in a reproducible manner and facilitate the comparison of results from different research laboratories. In conclusion we were unable to detect changes in lower extremity biomechanical or corticomotor response variables after a single session of anodal HD-tDCS to the ipsilesional M1 in chronic stroke. More work is required to determine how clinicians can best use neuromodulatory devices to improve lower extremity motor rehabilitation.

## Author Contributions

JK participated in data collection, analyzed and interpreted data, wrote first draft of the manuscript. SK provided conceptual framework of submitted work, critically revised manuscript for intellectual and scientific content. EW participated in data collection, critically revised manuscript for intellectual and scientific content. MB provided conceptual framework of submitted work, supervised all data collection, ensured adherence to ethical guidelines, critically revised manuscript for intellectual and scientific content, has final approval for manuscript submission.

## Conflict of Interest Statement

The authors declare that the research was conducted in the absence of any commercial or financial relationships that could be construed as a potential conflict of interest. The handling Editor declared a shared affiliation, though no other collaboration, with the authors.
